# The Publication Committee of the American Medical Association

**Published:** 1854-07

**Authors:** 


*The Publication Committee of the American Medical Association*—As we anticipated, the change in this committee occasions some discussion. Our readers, on referring to the report in our last number, will find that Prof. White, of this city, as chairman of the Nominating Committee, reported a majority of the Publication Committee in the city of New York; thus removing it from Philadelphia, where it had steadily remained from the organization of the Association. This was a step of considerable importance, and should not have been taken without just cause therefor. That such cause existed, we anticipate no difficulty in proving.

That the opposite side of the argument may be fairly presented, we subjoin the following remarks from Dr. Bulkley, of the New York Medical Times:

"The only occurrence which seems at all to have disturbed the harmony of the meeting, was the transfer of the Publication Committee from Philadelphia to this city; the discussion of which is said to have elicited some sectional and personal feeling, while the decision led to the offer of the resignation, by his colleagues, of Dr. Condie, as Treasurer of the Association. We regret this measure on many accounts, the more especially as it will have a tendency to mar the cordial feeling which should exist between the sister cities, and will be particularly unpleasant when we visit them, as many of us hope to do, next year, and partake of their hospitalities. The Association at first refused to accept the report of the Nominating Committee in favor of the change; but it was subsequently adopted, after a second and rather warm discussion in the committee of the whole. The change is said to have been made in accordance with the expressed wishes of the New York delegation; and if so, we cannot believe that they fairly represented the wishes of their constituents. At least, we have never heard of any expression to that effect, and know that the measure is regretted by some of the strong friends of the Association among us. We regard it as the more unfortunate from the fact that a resolution subsequently passed, that in future the printing for the year shall be executed at the city at which the meeting is held, a measure of questionable propriety, by the way, in its general application, but which, in the present case, if confirmed at the next meeting, will transfer it back to Philadelphia, after an interval of one year only. A change could then have been made if thought desirable by the majority; and thus the unpleasant feeling would have been spared at least until after our meeting in that city."

It may occasion some little surprise that a New York journal should thus be found in antagonism to the interests of its own city. With this, however, we have nothing to do.

We also regret that "anything should occur to mar the cordial feeling existing between the sister cities;" but we cannot see why our Philadelphia friends should have any feeling at this action of the Association. The duties of the Publication Committee are onerous; they have been inflicted on Philadelphia members for as long a term as is right or fair. If there is any honor or profit accruing from its duties, they have had a six years' tenure thereof, and should make no objections to a transfer of them to other recipients. We are confident that there is too much good sense in the profession of Philadelphia to allow this action to interfere with their usual friendship and hospitality to New York; at least, any such action on their part would show a very narrow spirit, which we do not think they possess.

The "Times" slightly mistakes the first action of the Association on the report of the Nominating Committee. The question of removal had been warmly discussed in committee. On the presentation of the majority report, it was desired by the minority to secure a discussion in open convention. This was effected by Dr. Reyburn, who moved the adoption of the report, "with the exception of that part which refers to the Committee on Publications." In this action of the Association, no rejection of the part named was implied. It was simply a concession, by the majority, of the privilege of prolonged debate. Previous to this, and after the adoption of the policy of removal in the Committee, the majority of that Committee moved a reconsideration, to remove a cause of complaint on the part of the minority, that the matter had been sprung upon them without due notice. This opened the way for a second debate in committee, which fully endorsed (and by an increased vote) its former action. The change was finally ordered by a nearly two-thirds vote of the Association. Whether or no "the change was made in accordance with the expressed wish of the New York Delegation," we do not know. As New York had but thirteen delegates in all, and inasmuch as each state was represented by a single vote in the Nominating Committee, it is probable that other delegations expressed a similar wish.

That in so acting they fairly represented their constituency, we have no doubt. We are fully aware of the feeling among the profession in this section, and, so far as we can judge, that feeling existed wide-spread. Few Journals were found to compliment the Publication Committee on their labors of the last year; and there was a very general complaint on the part of subscribers, on several grounds. If we chose, we could enumerate the causes of these complaints, and show that they were fully justified. We could repeat the protest we have already entered against the needless delay of the publication of the Transactions; of their shabby appearance when printed; of the appropriation of large sums to engravings of little worth; and the subsequent appropriation of these engravings to individual purposes and profit, without adequate remuneration to the Association. But we choose to place the justification of the Committee on other and less offensive grounds.

The continued publication of the Transactions in a single city, and the placing continued power in the hands of a single Committee, have had a tendency to confer upon a few persons too large a share in the control of the Association; and to bestow upon their city a factitious importance, as the center of medical operations in this country. While we acknowledge the industry and talent of the old Committee, and the prosperous condition of medical matters in their city, we do not think it right that the Association should become an instrument for the advancement of either, to the detriment of other towns.

Again, as we before remarked, there is no reason why Philadelphia should have any preference over New York. *Prima facie*, the profession of the latter city is as respectable as that of the former. We have yet to learn that New York is behind Philadelphia in medical talent, industry, or advantages; or that it lacks the mechanical facilities for the publication of the Transactions.

We repeat, that Philadelphia has no just cause for complaint in this matter, and we may add, that as no censure is implied in the action of the Association, they should cherish no revengeful spirit.

The "Times" is undoubtedly correct in deploring that subsequent action of the Association, providing for the future printing of the Transactions in the city where the yearly meeting may have been held. Such a plan would, in some instances, involve poor mechanical execution, and the resolution should be rescinded. But we rejoice that the Association has thus declared itself in favor of some system of rotation. If the system adopted at this meeting proves unserviceable, as we have no doubt it will, it will take but little action to frame a better plan.

Our own rivalries are far less with Philadelphia than New York. The latter city is nearest, and altogether most troublesome in its influence upon our interests. But we are glad to recognize in it only a spirit of honorable competition, with which we have no fault to find. H.

